# Gastroprotective and Healing Effects of *Polygonum cuspidatum* Root on Experimentally Induced Gastric Ulcers in Rats

**DOI:** 10.3390/nu12082241

**Published:** 2020-07-27

**Authors:** Young-Sik Kim, YunSeol Nam, Jungbin Song, Hocheol Kim

**Affiliations:** Department of Herbal Pharmacology, College of Korean Medicine, Kyung Hee University, 26 Kyungheedae-ro, Dongdaemun-gu, Seoul 02447, Korea; yjbsik@gmail.com (Y.-S.K.); yunseol@naver.com (Y.N.)

**Keywords:** *Polygonum cuspidatum*, gastric ulcer, gastritis, gastric acid, antioxidant, prostaglandin E_2_

## Abstract

*Polygonum cuspidatum* is widely used as food and medicine in Korea, China, and Japan. Its major bioactive components, resveratrol and emodin, reportedly protect against gastric lesions. We therefore aimed to investigate: (1) the gastroprotective effects of *P. cuspidatum* roots in hydrochloric acid/ethanol (HCl/EtOH)- and indomethacin-induced acute gastric ulcer rat models; (2) the healing effects in an acetic acid-induced ulcer model; and (3) potential mechanisms by measuring gastric acid secretion-related parameters in a pyloric ligation-induced ulcer model, and by measuring antioxidant enzyme and prostaglandin E_2_ levels in the gastric tissue of HCl/EtOH-treated rats. Oral administration of *P. cuspidatum* extract (PCE) at doses of 100 and 300 mg/kg significantly decreased HCl/EtOH- and indomethacin-induced gastric lesions. PCE at 300 mg/kg significantly reduced gastric lesions in acetic acid-induced ulcers. PCE increased superoxide dismutase (SOD) activity and glutathione (GSH) and prostaglandin E_2_ levels in gastric tissue, whereas it did not alter gastric acid secretion-related parameters. Our findings indicate that PCE has gastroprotective effects against HCl/EtOH and non-steroidal anti-inflammatory drugs (NSAIDs) and promotes healing of acetic acid-induced ulcers. These gastric mucosal protection and ulcer healing effects are associated with antioxidant effects and the augmentation of prostaglandin E_2_ and suggest that *P. cuspidatum* might be a promising preventive and therapeutic agent for treating gastric ulcers.

## 1. Introduction

Gastric ulcers result from damage to the mucosal lining of the stomach due to an increase in aggressive factors such as reactive oxygen species (ROS) and gastric acid, and a decrease in protective factors such as prostaglandins and mucus [1,2]. The most common symptom is upper abdominal pain, which occurs at night and is relieved by food intake [3]. Gastric ulcers are very painful and lower the quality of life of those who suffer from them [4]. Lack of proper gastric ulcer treatment can induce gastrointestinal bleeding, perforation, and gastric outlet obstruction [5]. In South Korea, about 10% of people have suffered from gastric ulcers [6]. Lifestyle habits, such as excessive alcohol intake or prolonged use of non-steroidal anti-inflammatory drugs (NSAIDs), are factors that cause gastritis and gastric ulcer [7]. Infection by the common bacteria *Helicobacter pylori* is another significant cause of gastric ulcers.

Gastric ulcers are characterized by chronicity due to repeated episodes of healing and re-exacerbation [8,9]. As this disease can persist for many years, a long-term management therapy for patients with a gastric ulcer is essential [10]. The primary treatment entails reducing the production of gastric acids with antacids, proton pump inhibitors, or H_2_ receptor antagonists [11]. In the case of a gastric ulcer associated with *H. pylori*, treatment consists of a combination of antibiotics and acid secretion inhibitors [12]. Despite good therapeutic efficacy, the long-term use of acid secretion inhibitors often causes side effects, such as osteoporosis and hypergastrinemia [13]. For these reasons, research into the development of gastroprotective agents that are safer for long-term use, and which protect the gastric mucosa without affecting acid secretion, have attracted interest. Natural products have long been known to be safe and effective in treating gastric ulcer [7].

*Polygonum cuspidatum* Sieb. et Zucc., commonly called Asian or Japanese knotweed, is an herbaceous perennial plant native to East Asia. The stem and root of this plant are widely used as food and medicine in Korea, China, and Japan [14,15]. In particular, the root of *P. cuspidatum* has long been used for the treatment of pain, inflammation, infection, skin burns, scalding, and jaundice [14,16,17]. The major active constituents are anthraquinones (emodin, physcion, anthraglycoside B, etc.) and stilbenes (polydatin, resveratrol, etc.), which are known to be responsible for the diverse pharmacological properties of *P. cuspidatum* [14,18,19,20].

Recently, resveratrol and emodin, the major bioactive components of *P. cuspidatum*, have been reported to protect against gastric lesions. Resveratrol shows gastroprotective, anti-inflammatory, and antioxidant effects on EtOH-induced gastritis in rats [21], and also showed gastroprotective and ulcer healing effects on acetic acid-induced gastric ulcer [22]. Emodin was shown to protected against gastric ulcers induced by cold restraint, alcohol, aspirin, and pyloric ligation by reducing gastric acid secretion and increasing mucin and prostaglandin E_2_ (PGE_2_) secretion [23]. These previous findings led us to hypothesize that *P. cuspidatum* may have gastroprotective and ulcer healing effects. This study therefore aimed to investigate these effects using *P. cuspidatum* extract (PCE), and to explore the underlying mechanisms of these effects using different gastric ulcer rat models. Gastric ulcers were induced by hydrochloric acid/ethanol (HCl/EtOH), indomethacin, and acetic acid; lesion areas were then measured. To determine the effect of *P. cuspidatum* on the aggressive and protective factors that are involved in gastric ulcer, we measured gastric secretion parameters in pylorus-ligated rats and evaluated the levels of antioxidant factors and PGE_2_ in the gastric tissues of HCl/EtOH-treated rats.

## 2. Materials and Methods

### 2.1. Plant Materials and Sample Preparation

Dried *P. cuspidatum* roots were obtained from Daewoo Medicinal Herbs Co. (Seoul, Korea). The plant materials were authenticated by professor Hocheol Kim of Kyung Hee University and extracted with 10 volumes of 30% EtOH at 85 °C for 3 h in a reflux apparatus. The extract was concentrated in vacuo using a rotary evaporator (Eyela N-1000, Tokyo, Japan), and lyophilized in a freeze dryer (Operon OPR-FD4-8612, Gyeonggi-do, Korea). The final extract yield was 15.53%. The extract was kept in the refrigerator at 4 °C. The voucher plant material specimens were deposited in the Department of Herbal Pharmacology, College of Korean Medicine, Kyung Hee University.

### 2.2. High-Performance Liquid Chromatography (HPLC) Analysis

The contents of the marker compounds, polydatin and emodin, were quantified with HPLC on an Agilent 1220 HPLC system (Agilent Technology, Santa Clara, CA, USA) that was equipped with a G4281B binary pump, a G4282B autosampler, and a G4285B diode array detector using a Sunfire™ C18-column (5 µm; 250 × 4.6 mm; Waters, Milford, MA, USA). The mobile phase consisted of 0.1% (*v/v*) phosphoric acid in distilled water (A) and acetonitrile (B). The gradient elution conditions for detection were as follows: 0–60–65–67–72 min, 0–100–100–0–0% solvent B. The flow rate was 1.0 mL/min. The detection wavelengths were set to 306 nm for polydatin and resveratrol and to 290 nm for emodin, according to the maximum absorption wavelength of each compound.

### 2.3. Animals

Six-week-old male Sprague-Dawley (SD) specific pathogen-free rats and seven-week old male Wistar rats (Samtako, Gyeonggi-do, Korea) were used after a 1-week acclimatization period. Rats were housed under standard laboratory conditions with 23 ± 1 °C temperature, 55 ± 5% relative humidity, and 12 h light/dark cycle and allowed free access to food and water. Rats were monitored once daily for health status during the acclimatization period; no abnormal findings were observed. All experimental procedures were approved by the Institutional Animal Care and Use Committee, Korea Institute of Science and Technology for Eastern Medicine (no. KISTEM-IACUC-2015-005) and Kyung Hee University Institutional Animal Care and Use Committee (no. KHUASP (SE)-17-081).

### 2.4. HCl/EtOH-Induced Acute Gastric Lesions

The HCl/EtOH-induced ulceration was conducted according to the protocol of Mizui and Doteuchi [24]. Thirty SD rats were randomly assigned to four groups: control (*n* = 8), omeprazole 20 mg/kg (positive control, *n* = 7), PCE 100 mg/kg (*n* = 7), and PCE 300 mg/kg (*n* = 8). After a 24 h fast, samples were administered by oral gavage at a volume of 10 mL/kg body weight; the control group received the vehicle (distilled water) at the same volume. One hour after sample administration, 1.5 mL of 60% EtOH in 150 mM HCl solution was administered by oral gavage for induction of gastric lesions. The rats were euthanized by cervical dislocation 1 h post-HCl/EtOH administration. The stomachs were quickly removed, incised along the greater curvature, and then washed with sterile saline. The stomachs were stretched on a flat plate and photographed to analyze the gastric lesions. The gastric samples were stored at −80 °C until used for analysis of antioxidant enzyme activity and PGE_2_ concentration. The area of gastric lesion was measured using image J program (National Institutes of Health, Bethesda, Rockville, MD, USA). The gastric lesion index was calculated as follows:

$$Gastric\;lesion\;index=\frac{Gastric\;lesion\;area\;\left(mm^{2}\right)}{Total\;gastric\;area\;\left(mm^{2}\right)}\times 100$$

### 2.5. Indomethacin-Induced Acute Gastric Lesions

The effects of PCE on indomethacin-induced gastric lesions were assessed, and the gastroprotective effects of PCE were compared with those of omeprazole, a proton pump inhibitor. Twenty-eight SD rats were randomly assigned to four groups: control (*n* = 8), omeprazole 20 mg/kg (*n* = 6), PCE 100 mg/kg (*n* = 7), and PCE 300 mg/kg (*n* = 7). After a 24 h fast, samples were administered by oral gavage at a volume of 10 mL/kg body weight; the control group received the vehicle (distilled water) at the same volume. Thirty minutes after sample administration, indomethacin in 5% NaHCO_3_ solution was administered orally at a dose of 100 mg/kg for the induction of gastric lesions. The rats were euthanized by cervical dislocation 6 h post indomethacin administration. The stomachs were quickly removed, incised along the greater curvature, and then washed with sterile saline. The stomachs were stretched on a flat plate and photographed to analyze the gastric lesions. The area of gastric lesion was measured using image J program (National Institutes of Health, Bethesda, USA). The gastric lesion index was calculated as described above.

### 2.6. Acetic Acid-Induced Chronic Gastric Lesions

Chronic gastric lesions were induced as described by Okabe et al. [25]. After a 24 h fast, 28 Wistar rats were anesthetized with 2% isoflurane in 70% nitrous oxide and 30% oxygen. After each abdomen was opened by small midline incision and the stomach was slightly lifted out, 500 μL of 100% acetic acid was instilled into a 6 mm diameter plastic cylinder and allowed to remain on the serosa of the stomach for 1 min. After aspiration of acetic acid, the serosa was washed with saline, the stomach was replaced carefully into the abdomen, and then the abdominal incision was closed. After surgical recovery, the 28 rats were randomly assigned to three groups: control (*n* = 10), omeprazole 20 mg/kg (*n* = 10), and PCE 300 mg/kg (*n* = 8). Beginning two days after ulcer induction, samples were administered by oral gavage at a volume of 10 mL/kg body weight twice a day for a week, and the control group received the vehicle (distilled water) at the same volume. At the end of the treatment period, the rats were euthanized by cervical dislocation and the stomachs were quickly removed. The gastric ulcer lesion area and index were determined as described above.

### 2.7. Gastric Secretion in Pylorus-Ligated Rats

The free and total acidity, volume, and pH of the gastric juice were determined using the method of Shay and Gruenstein [26]. Twenty-six SD rats were randomly assigned to four groups: control (*n* = 9), omeprazole 20 mg/kg (*n* = 6), PCE 100 mg/kg (*n* = 6), and PCE 300 mg/kg (*n* = 5). After a 24 h fast, samples were administered by oral gavage at a volume of 10 mL/kg body weight; the control group received the vehicle (distilled water) at the same volume. After 30 min of sample administration, rats were anesthetized with 2% isoflurane in 70% nitrous oxide and 30% oxygen. A 2 cm midline incision was made below the xiphoid process, and then the pyloric portion of the stomach was slightly lifted out and ligated avoiding bleeding or blood vessel occlusion. The stomach of each rat was placed carefully back into the abdomen and the wound was sutured. After 4 h of pylorus ligation, rats were euthanized by cervical dislocation and the stomach was removed. The gastric juice was collected and centrifuged at 3000 rpm for 10 min. The volume and pH of gastric juice were measured using a measuring cylinder and a pH meter (Ultrabasic benchtop meters, Denver Instrument, Denver, CO, USA). Free acidity was quantified by titration with 0.05 N sodium hydroxide using phenolphthalein as an indicator. Total acidity was calculated as follows:

Total Acidity (mEq/4hr)=Vol. of gastric juice (mL)×Vol. of NaOH (mL)×normality of 0.05N NaOH

### 2.8. Measurement of Superoxide Dismutase (SOD), Glutathione (GSH) Level, and Catalase (CAT) Activity

The gastric antioxidant enzyme activity was measured in the HCl/EtOH-induced gastric ulcer model. Frozen stomach tissues were pulverized with liquid nitrogen using a mortar and pestle. The ground tissues (about 200 mg) were homogenized using a homogenizer (Bead Ruptor 24 Elite, OMNI International, Kennesaw, GA, USA) in 1 mL of 50 mM sodium phosphate buffer (pH 7.0) containing 1 mM ethylenediaminetetraacetic acid (EDTA). Homogenates were centrifuged at 4000 rpm for 5 min at 4 °C and the supernatant was divided into two portions; one was used to measure SOD activity, and the other was centrifuged at 10,000 rpm for 15 min at 4 °C in order to measure the CAT and GSH activities of the resultant supernatant. The activities of SOD, CAT, and GSH were determined using a SOD assay kit (catalog no. 706002, Cayman Chemical Company, Ann Arbor, MI, USA), a CAT assay kit (catalog no. 707002, Cayman Chemical Company, USA), and a GSH assay kit (catalog no. 703002, Cayman Chemical Company, USA), respectively, according to the manufacturers’ protocols. The absorbance was measured at 450, 540, and 410 nm, respectively, using a microplate spectrophotometer (Epoch 2, BioTek, Winooski, VT, USA). The antioxidant enzyme activity was normalized to the wet gastric tissue weight.

### 2.9. Determination of PGE_2_

In order to evaluate the concentration of PGE_2_ in stomach tissue obtained from the HCl/EtOH-induced gastric ulcer model, about 200 mg of stomach tissue was pulverized with liquid nitrogen using a mortar and pestle, then homogenized in 0.5 mL of 100 mM phosphate buffer (pH 7.4) containing 1 mM EDTA and 10 μM indomethacin. Homogenized gastric samples were centrifuged at 8000 rpm for 10 min at 4 °C; the PGE_2_ concentration in the supernatant was quantified using a PGE_2_ express enzyme immunoassay kit (catalog no. 500141, Cayman Chemical Company, USA) according to the manufacturer’s protocol. The absorbance was measured using a microplate reader (Epoch 2, BioTek, USA). The PGE_2_ concentrations were normalized to the wet gastric tissue weight, and the results are expressed as pg/mg wet tissue.

### 2.10. Statistical Analysis

Values are presented as mean ± the standard deviation. Statistical analysis was performed using GraphPad Prism 8 (GraphPad Software Inc., La Jolla, San Diego, CA, USA). Shapiro–Wilk test and Bartlett’s test were used to determine normality and homoscedasticity, respectively. One-way analysis of variance (ANOVA) with Dunnett’s test was used to analyze the normal, homoscedastic data. For heteroscedastic data, Welch’s ANOVA with Dunnett’s T3 multiple comparisons test or Kruskal–Wallis test with Dunn’s multiple comparisons test (a non-parametric method) were used to test significance. *p* < 0.05 was considered statistically significant.

## 3. Results

### 3.1. HPLC Analysis of PCE

**Figure 1:**
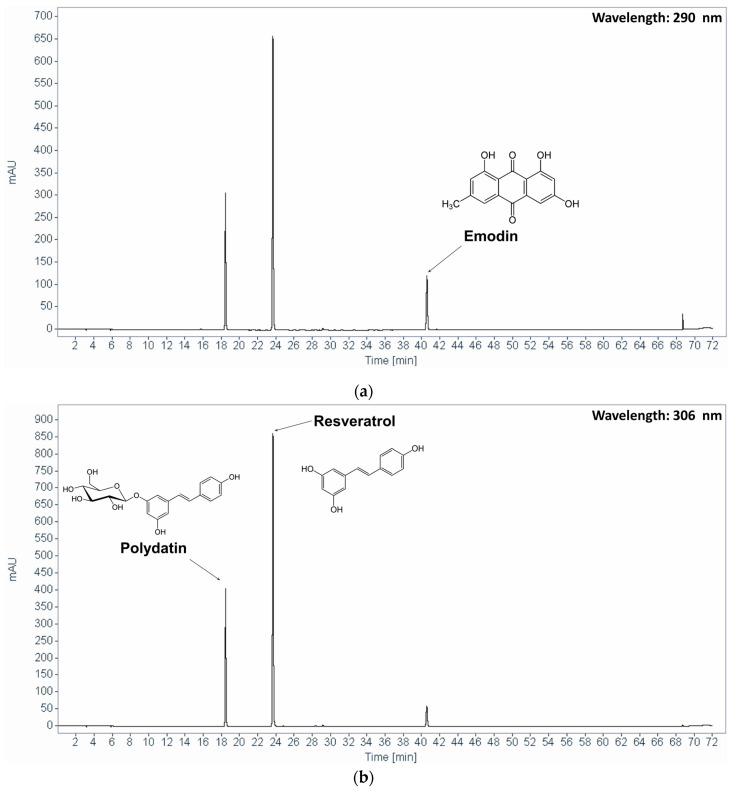

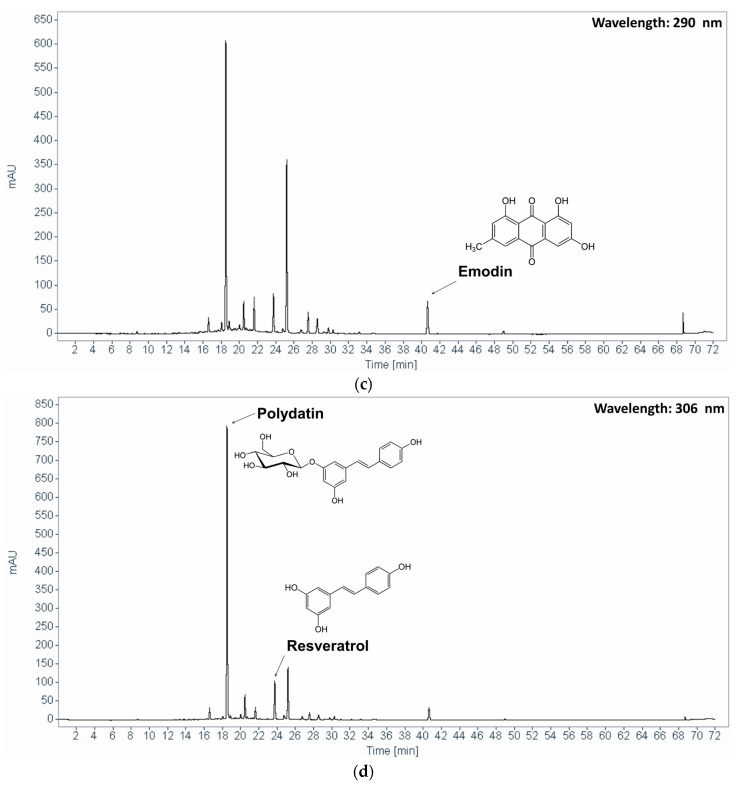
Chromatograms of (**a**) standard mixture at 290 nm, (**b**) standard mixture at 306 nm, (**c**) PCE at 290 nm, (**d**) PCE at 306 nm. PCE, *P. cuspidatum* extract.

Figure 1 shows the representative HPLC chromatograms of the standards mixture and PCE. The polydatin, resveratrol, and emodin contents were 8.66 mg/g, 2.90 mg/g, and 3.95 mg/g, respectively.

### 3.2. Effect of PCE on HCl/EtOH-Induced Acute Gastric Lesions

**Figure 2:**
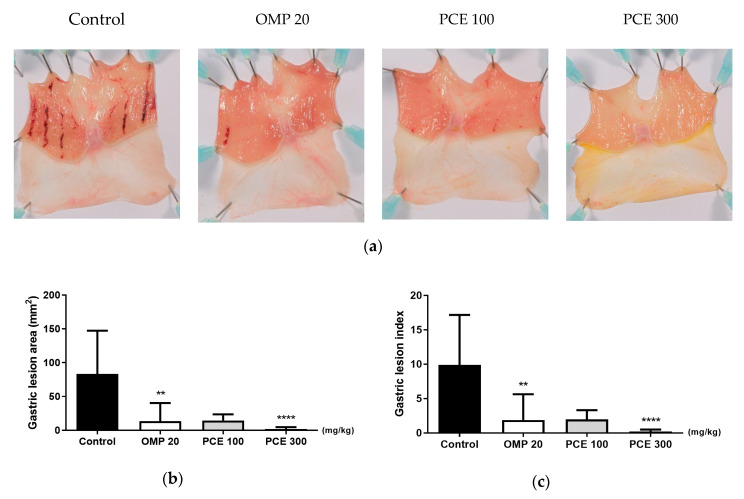
Effects of PCE on hydrochloric acid/ethanol (HCl/EtOH)-induced rat gastric mucosal injury. (**a**) Representative macroscopic images of stomachs from each group. One hour prior to HCl/EtOH administration, rats were orally treated with distilled water (control), OMP 20 mg/kg, PCE 100 mg/kg, or PCE 300 mg/kg. Measurement of gastric lesion area (**b**) and gastric lesion index (**c**). Values are expressed as mean ± standard deviation. *n* = 7–8 per group. ** *p* < 0.01 and **** *p* < 0.0001 vs. control by Kruskal–Wallis test with Dunn’s multiple comparisons test. OMP, omeprazole; PCE, *P. cuspidatum* extract.

HCl/EtOH administration caused severe hemorrhagic lesions that appeared as elongated bands parallel to the long axis of the glandular stomach in control rats (Figure 2a). Whereas the control group showed a lesion area of 83.3 ± 64.0 mm^2^, the lesion areas of rats treated with PCE at doses of 100 and 300 mg/kg were reduced to 14 ± 9.2 mm^2^ (not significant) and 1.7 ± 2.9 mm^2^ (*p* < 0.0001), respectively (Figure 2b). The positive control, omeprazole (20 mg/kg), also significantly reduced the lesion area to 13.5 ± 26.7 mm^2^ (*p* < 0.01). A significant decrease in gastric lesion index concomitant with the reduction in lesion area was observed for the PCE (300 mg/kg) and omeprazole treatments that were administered before HCl/EtOH induction (Figure 2c).

### 3.3. Effect of PCE on Indomethacin-Induced Acute Gastric Lesions

**Figure 3:**
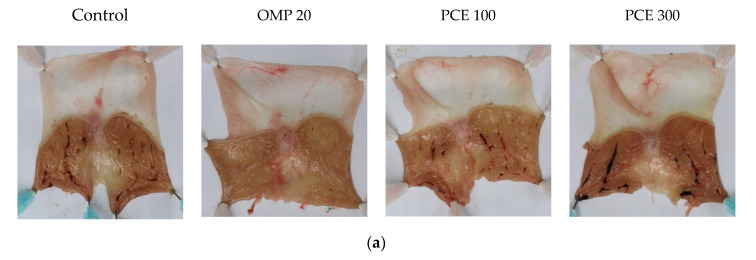

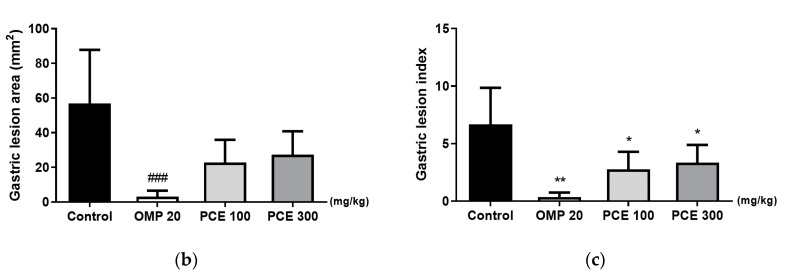
Gastroprotective effects of PCE on indomethacin-induced ulcer in the gastric mucosa of SD rats. (**a**) Representative macroscopic images of stomachs from each group. Rats were pre-treated orally with distilled water (control), OMP 20 mg/kg, PCE 100 mg/kg, or PCE 300 mg/kg 30 min prior to indomethacin administration. Measurement of gastric lesion area (**b**) and gastric lesion index (**c**). Values are expressed as mean ± standard deviation. *n* = 6–8 per group. * *p* < 0.05, ** *p* < 0.01 vs. control by Welch’s analysis of variance (ANOVA) with Dunnett’s T3 multiple comparisons test, ^###^ *p* < 0.001 vs. control by Kruskal–Wallis test with Dunn’s multiple comparisons test. OMP, omeprazole; PCE, *P. cuspidatum* extract.

Figure 3a shows that indomethacin administration induced hemorrhagic gastric lesions in the glandular stomach. Omeprazole significantly reduced the gastric lesion area to 3.1 ± 3.4 mm^2^ (*p* < 0.001), compared to the control group, which showed a gastric lesion area of 56.8 ± 30.9 mm^2^ (Figure 3b). There were no statistically significant differences in the gastric lesion area between the PCE and control groups. The gastric lesion index showed that treatment with PCE at doses of 100 and 300 mg/kg and omeprazole at 20 mg/kg significantly reduced the gastric lesion index from 6.4 ± 2.8 in the control group to 2.8 ± 1.5 (*p* < 0.05), 3.3 ± 1.5 (*p* < 0.05), and 0.4 ± 0.4 (*p* < 0.01), respectively (Figure 3c).

### 3.4. Healing Effect of PCE on Acetic Acid-Induced Chronic Gastric Ulcer

**Figure 4:**
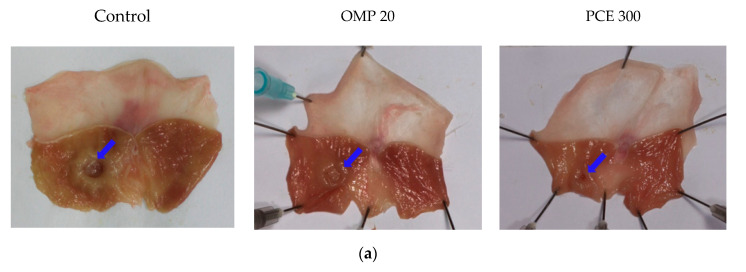

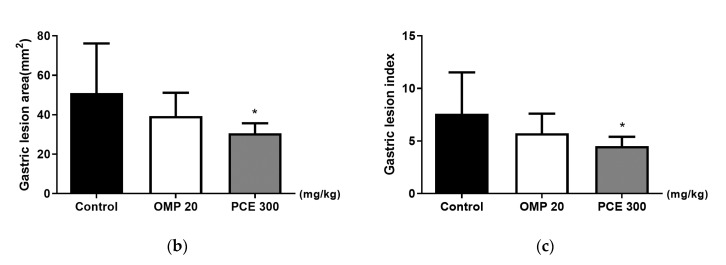
Healing effects of PCE on gastric ulcers induced by acetic acid. (**a**) Representative macroscopic images of stomachs from each group. Rats were treated orally twice a day with distilled water (control), OMP (20 mg/kg), or PCE (300 mg/kg) after surgery. Quantification of gastric lesion area (**b**) and gastric lesion index (**c**). Values are expressed as mean ± standard deviation. *n* = 8–10 per group. * *p* < 0.05 vs. control by Kruskal–Wallis test with Dunn’s multiple comparisons test. OMP, omeprazole; PCE, *P. cuspidatum* extract.

The high dose (300 mg/kg) of PCE was selected for the acetic acid gastric ulcer model, as it was more effective than the low dose (100 mg/kg) in reducing HCl/EtOH-induced gastric lesions. The instillation of acetic acid resulted in chronic gastric ulcer in control rats, whereas the wounds of PCE-treated rats were markedly healed (Figure 4a; indicated by blue arrows). Treatment with PCE at 300 mg/kg significantly reduced the gastric lesion area (30.6 ± 5.1 mm^2^, *p* < 0.05) and gastric lesion index (4.5 ± 0.9, *p* < 0.05) compared to the control group (51.0 ± 25.2 mm^2^ and 7.6 ± 3.9, respectively; Figure 4b,c). Omeprazole treatment did not significantly reduce gastric lesion area and index compared to the control rats.

### 3.5. Effect of PCE on Gastric Secretion

In pylorus-ligated rats, PCE treatment did not affect the gastric secretion parameters, including volume, pH, and acidity of gastric juice (Table 1). Omeprazole significantly increased the pH value and decreased free acidity compared to the control group.

### 3.6. Effects of PCE on the Levels of SOD, GSH, and CAT in the Stomach Tissue of the HCl/EtOH-Treated Rats

**Figure 5:**
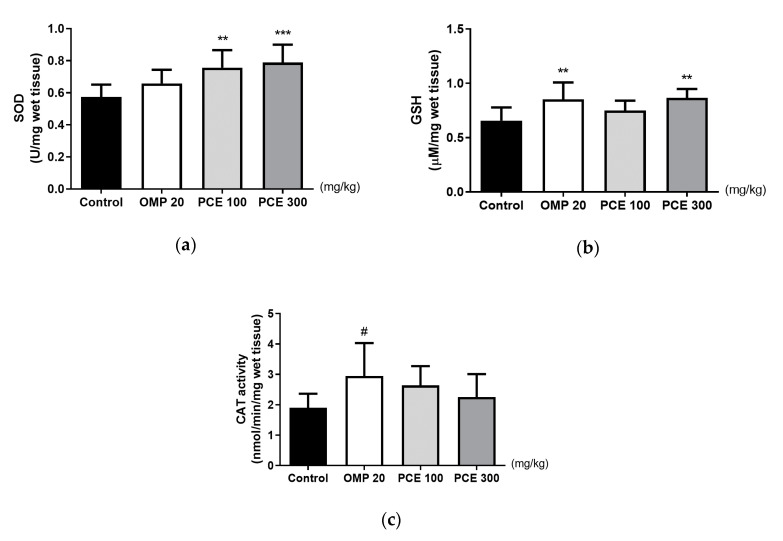
Effects of PCE on the levels of (**a**) superoxide dismutase (SOD), (**b**) glutathione (GSH), and (**c**) catalase (CAT) activity in the stomach tissue of the hydrochloric acid/ethanol (HCl/EtOH)-treated rats. Rats were pre-treated orally with distilled water (control), OMP 20 mg/kg, PCE 100 mg/kg, or PCE 300 mg/kg 1 h prior to HCl/EtOH administration. Values are expressed as mean ± standard deviation. *n* = 7–8 per group. ** *p* < 0.01, *** *p* < 0.001 vs. control by one-way analysis of variance (ANOVA) with Dunnett’s post-hoc test. ^#^ *p* < 0.05 vs. control by Kruskal–Wallis test with Dunn’s multiple comparisons test. OMP, omeprazole; PCE, *P. cuspidatum* extract.

The SOD levels were significantly higher in the stomach tissue of HCl/EtOH-treated rats that had received PCE at doses of 100 and 300 mg/kg than in the control group (*p* < 0.01 and *p* < 0.001, respectively; Figure 5a). The GSH level was also significantly higher in the PCE 300 mg/kg group than in the control group, but CAT activity was not altered by PCE treatment (Figure 5b,c). Omeprazole significantly increased GSH level and CAT activity compared to the control group but did not change SOD level.

### 3.7. Effects of PCE on the PGE_2_ Level of Stomach Tissue

**Figure 6:**
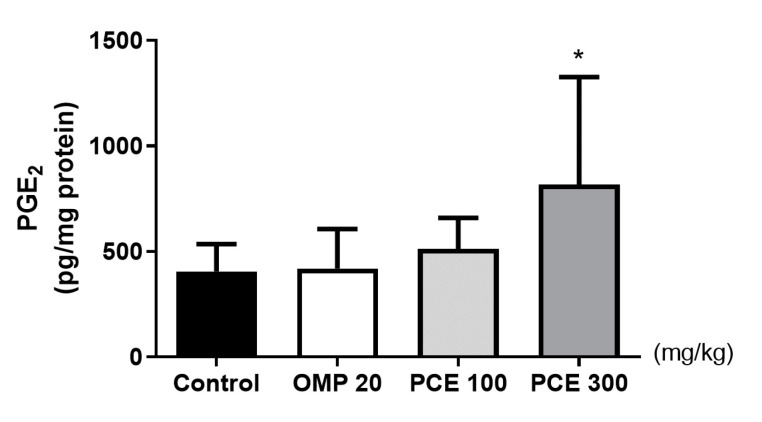
Effects of PCE on the levels of prostaglandin E_2_ (PGE_2_) in the stomach tissue of the hydrochloric acid/ethanol (HCl/EtOH)-treated rats. Rats were pre-treated orally with distilled water (control), OMP 20 mg/kg, PCE 100 mg/kg, or PCE 300 mg/kg 1 h prior to HCl/EtOH administration. Values are expressed as mean ± standard deviation. *n* = 7–8 per group. * *p* < 0.05 vs. control by Kruskal–Wallis test with Dunn’s multiple comparisons test. OMP, omeprazole; PCE, *P. cuspidatum* extract.

The stomach tissue PGE_2_ level of HCl/EtOH-treated rats that received PCE at a dose of 300 mg/kg was significantly higher than that of the vehicle group (*p* < 0.05, Figure 6), whereas no significant difference was observed in the omeprazole group.

## 4. Discussion

We first demonstrated that PCE pretreatment prevents acute gastric mucosal injury induced by both HCl/EtOH and indomethacin, while PCE post-treatment promotes the healing of acetic acid-induced chronic gastric lesions. In addition, PCE increased SOD activity and GSH and PGE_2_ levels in rat gastric tissue after HCl/EtOH administration.

The gastric mucosa is the first protective layer of gastric tissue, and the mucus gel of the gastric mucosa prevents the diffusion of digestive enzymes into the stomach wall [27]. This protective layer is damaged by EtOH, which causes erosion, ulcers, and petechial hemorrhages in the gastric mucosa by producing ROS, peroxide anions, and hydroperoxyl free radicals. Co-treatment with HCl evokes rapid and severe gastric injury [28]. Therefore, HCl/EtOH-induced gastric ulcers are considered a reliable tool for evaluating the efficacy of gastroprotectants. Pretreatment with PCE at doses of 100 and 300 mg/kg significantly reduced gastric lesions by 82.8% and 97.9%, respectively, in rats treated with HCl/EtOH; its gastroprotective effect is comparable to that of omeprazole. These results indicate that PCE pretreatment has gastroprotective effects against acute mucosal damage caused by HCl/EtOH.

Gastric ulcers are a well-recognized complication of NSAID use. Among the widely used NSAIDs, indomethacin has the strongest ulcerogenic effect in humans and is the most commonly used ulcerogen in gastric ulcer induction [8]. Inhibition of cyclo-oxygenases (COXs) and the subsequent decrease in prostaglandin synthesis are known to be the major mechanisms contributing gastric pathogenesis induced by NSAIDs such as indomethacin [29]. In this study, pretreatment with PCE prevented indomethacin-induced acute mucosal damage in rats, suggesting that PCE exerts gastroprotective effects against NSAID-induced acute gastric ulcers.

One of the major challenges for physicians and ulcer patients is the chronic nature of gastric ulcers, which is represented by repeated cycles of healing and re-exacerbation. Experimentally induced ulcerative lesions mostly heal within a few days without scarring and do not recur spontaneously [30]. On the other hand, acetic acid causes chronic gastric ulcer because of its long-term persistence. The spontaneous recurrence of healed ulcers in this model is similar to human chronic ulcers; thus, the acetic acid model used in this study is standard for screening medicines as potential ulcer healing drugs [8,25]. The mechanisms associated with ulcer healing include increased antioxidant activity, PGE_2_ generation_,_ anti-inflammation factors, inhibition of gastric acid secretion, angiogenesis, increased gastric mucus secretion, and the presence of growth factors [25,31,32]. Resveratrol, the major component of PCE, has been shown to reduce the ulcer index in acetic acid-induced gastric ulcers [22]. Our study shows that seven days of PCE treatment after ulcer induction with acetic acid reduced gastric lesions, whereas a comparable treatment regimen with omeprazole did not. This suggests that the healing effects of PCE that we observed in acetic acid-induced ulcer may be partially attributable to resveratrol.

The gastric mucosa maintains its function and structural integrity through complex, multifactorial interactions between protective factors (including mucin, prostaglandin, and antioxidants) and aggressive factors (such as gastric acid and pepsin) [33,34]. Under normal conditions, protective factors prevent local damage from continuous exposure to ulcerogens and maintain structural and functional mucosal integrity [35]. Gastric ulcers caused by EtOH are associated with mass production of ROS, which can provoke oxidative damage [36]. Antioxidants including SOD, CAT, and GSH can scavenge oxygen-derived free radicals in the gastric mucosa [37]. Depletion of cellular GSH levels, as well as reduced SOD and CAT activities, impedes recovery shortly after EtOH-induced gastric oxidative injury [38,39,40]. Increases in SOD, GSH, and CAT levels of the gastric tissues contribute to gastroprotection against indomethacin and to the healing of acetic acid-induced gastric mucosal injury [41,42,43]. Resveratrol, a main active component of *P. cuspidatum*, has been reported to produce gastroprotective and ulcer healing effects in EtOH- and acetic acid-induced ulcer models, respectively, by augmenting SOD, GSH, and CAT levels [21,22]. We found that the SOD activities and GSH levels were significantly higher in the gastric tissues of HCl/EtOH-ulcerated rats. These results suggest that the gastroprotective and ulcer healing effects of PCE might be partially mediated by the antioxidant effects of resveratrol.

Pretreatment with PCE significantly increased the PGE_2_ level in the gastric tissue of HCl/EtOH-ulcerated rats. Prostaglandins produced from arachidonic acid, mainly via COX-2 enzyme activity, are present throughout the gastrointestinal tract. PGE_2_ plays a particularly important role in gastric mucosal protection and ulcer healing by controlling gastric secretions of acid and bicarbonate, gastric blood flow, mucus production, and gastric mucosal integrity [44]. In particular, PGE_2_ has been reported to promote dilation and therefore blood flow of mucosal microvasculature [45]. These changes help flush free radicals from vicinity of epithelial cells and neutralize gastric acid, thus providing gastroprotection [46]. Experimental studies have shown that administration of PGE_2_ protects gastric mucosa against EtOH- and indomethacin-induced gastric injury and promotes healing of acetic acid-induced gastric ulcer. In addition, a previous study reported that emodin, the major component of PCE, increases PGE_2_ levels in aspirin-induced acute gastric ulcer [23]. Our results, along with previous findings, indicate that an increase in PGE_2_ levels might contribute to the gastroprotective and ulcer healing effects of PCE and account for its antioxidant properties.

PCE treatment did not affect gastric acid secretion in pylorus-ligated rats. Since pylorus ligation causes accumulation of gastric acid and pepsin in rats, this model is used for assessing gastric secretion [47]. Acid suppressing drugs such as proton pump inhibitors and H_2_-receptor antagonists are not considered gastric mucosal protective agents because they rely on acid secretion inhibition [48]. Furthermore, extended inhibition of acid secretion can increase susceptibility to infection and increase the risk of gastrointestinal cancer [48,49]. We therefore conclude that gastric acid suppression is not likely to be a mechanism underlying the beneficial effects of PCE.

This study utilized HCl/EtOH and indomethacin-induced rat gastric ulcer models, which are analogous to gastric mucosal damage caused by alcohol consumption and excessive NSAID use, respectively. Since *H. pylori* infection is also a major cause of gastric ulcers [7], a future study is warranted to investigate the effects of PCE in *H.* *pylori*-induced ulceration. Furthermore, because PCE treatment was associated with increased PGE_2_ levels in ulcerated gastric tissue, we posit that it increases mucosal microvascular blood flow. We propose to investigate the effects of PCE on microvascular blood flow in future studies.

## 5. Conclusions

In conclusion, *P. cuspidatum* shows gastroprotective and ulcer healing effects, which are likely associated with the antioxidant effects and the augmentation of PGE_2_. Based on these findings, *P. cuspidatum* might be a potential preventive and therapeutic agent for the treatment of gastric ulcers.

## Figures and Tables

**Table 1:** Effects of PCE on gastric secretion parameters in pylorus-ligated rats.

Groups	Gastric Volume (mL)	Gastric pH	Free Acidity (mEq/mL)	Total Acidity (mEq/mL)
Control	5.99 ± 1.64	1.68 ± 0.18	8.00 ± 2.86	13.72 ± 2.31
OMP 20 mg/kg	4.30 ± 0.99	3.07 ± 1.42 *	2.33 ± 2.66 ^##^	9.58 ± 3.98
PCE 100 mg/kg	4.52 ± 1.85	1.87 ± 0.60	7.08 ± 3.38	13.83 ± 2.40
PCE 300 mg/kg	6.24 ± 1.50	1.59 ± 0.15	9.40 ± 1.95	14.00 ± 2.32

Rats were pre-treated orally with distilled water (control), OMP 20 mg/kg, PCE 100 mg/kg, or PCE 300 mg/kg 30 min before pylorus ligation. Gastric secretion parameters were measured 4 h after pylorus ligation. Values are expressed as mean ± standard deviation. *n* = 5–9 per group. * *p* < 0.05 vs. control by Kruskal–Wallis test with Dunn’s multiple comparisons test, ^##^ *p* < 0.01 vs. control by one-way analysis of variance (ANOVA) with Dunnett’s post-hoc test. OMP, omeprazole; PCE, *P. cuspidatum* extract.
